# Transition to adolescence in neurodevelopmental disorders: challenges and clinical perspectives

**DOI:** 10.1016/j.jped.2025.101463

**Published:** 2025-10-24

**Authors:** Eduardo Jorge Custódio da Silva, Evelyn Eisenstein

**Affiliations:** a Universidade Federal do Rio de Janeiro (UFRJ), Neurologia, Rio de Janeiro, RJ, Brazil; b Instituto Nacional de Saúde da Mulher, da Criança e do Adolescente Fernandes Figueira (IFF/FIOCRUZ), Saúde da Criança, Rio de Janeiro, RJ, Brazil; c Faculdade de Ciências Médicas da Universidade do Estado do Rio de Janeiro (FCM-UERJ), Departamento de Pediatria, Rio de Janeiro, RJ, Brazil; d Sociedade Brasileira de Pediatria (SBP), GT sobre Saúde na Era Digital, Rio de Janeiro, RJ, Brazil; e Academia de Medicina do Rio de Janeiro (AMRJ), Rio de Janeiro, RJ, Brazil; f Clínica de Adolescentes e do Centro de Estudos Integrados da Infância, Adolescência e Saúde, Rio de Janeiro, RJ, Brazil

**Keywords:** Autism spectrum disorder (ASD), ADHD, Neurodevelopment, Adolescence, Adolescent health

## Abstract

**Objective:**

This review article describes the main clinical, emotional, educational, and social aspects involved in the transition to adolescence for individuals with these conditions. It also addresses implications for healthcare, the continuity of educational support, and the importance of individualized transition strategies, highlighting the need for a multidisciplinary approach centered on the individual and on continuity of care.

**Data sources:**

Review of the latest literature on neurodevelopmental disorders in adolescents.

**Data summary:**

Adolescence represents a vital period of biopsychosocial transformation, particularly challenging for individuals with neurodevelopmental disorders (NDDs), such as autism spectrum disorder (ASD), attention-deficit/hyperactivity disorder (ADHD), and intellectual disability.

**Conclusions:**

Integrated, person-centered, continuous, and collaborative health care is essential to promote the well-being and inclusion of these adolescents.

## Introduction

Neurodevelopmental disorders (NDDs) are childhood-onset conditions characterized by deficits in cognitive, behavioral, and social development, including autism spectrum disorder (ASD), attention deficit disorder (ADHD), language and learning disorders, and intellectual disability [1].

The number of cases of children and adolescents with NDDs, also known as neurodivergent disorders, diagnosed in Brazil and worldwide has increased significantly over the past few decades. The causes of this increase are still not fully understood; however, it is known that much of it is due to the increased knowledge of these conditions among professionals in various health fields, allowing for the correct diagnosis of previously unrecognized cases and the identification of milder cases.

These disorders are currently the most well-known and highly visible conditions that affect the brain and mental and behavioral development during childhood and adolescence. Today, few people have never heard of someone with these disorders or don’t have a history of someone with them. In this context, understanding neurodevelopmental disorders is crucial for care for neurodivergent individuals to be based on solid scientific evidence. The vulnerability of these populations, coupled with families' anguish in seeking quick solutions, can lead to adherence to therapies without pediatric technical support. Using a treatment without scientific evidence can lead to significant losses — not just financial ones — because, when deciding to pursue alternative approaches, many families end up abandoning interventions with proven efficacy. This decision can expose the patient to avoidable side effects, compromise therapeutic outcomes, and generate unnecessary expenses.

The transition from childhood to adolescence in these patients represents a critical point for diagnostic reassessment, adaptation of therapeutic strategies, and redefinition of functional goals.

Adolescents with NDDs face increased risks of social isolation, school difficulties, internalizing symptoms, emotional dysregulation, and mental health problems [2,3]. Furthermore, Brazilian health services are rarely prepared to provide continuous and integrated care that encompasses this stage of development [4].

## Neurobiological and psychosocial changes in adolescence

Adolescence is marked by significant neurohormonal changes in the brain that occur during puberty, especially in the prefrontal, limbic, and pituitary regions. These changes affect hormonal and emotional regulation, inhibitory control, and decision-making, as well as bodily, genital, and sexual growth [5]. In individuals with NDDs, these changes can exacerbate existing symptoms or contribute to the emergence of new clinical manifestations, such as anxiety, depression, or risky behaviors [6].

From a psychosocial perspective, the challenges of developing autonomy and independence, identity, and sexuality take on distinct contours. Many adolescents with ASD, for example, report difficulties interpreting social norms and establishing emotional bonds [7].

## Specificities of neurodevelopmental disorders

### Autism spectrum disorder (ASD)

During adolescence, individuals with ASD may experience worsening symptoms of irritability, increased stereotypies, or the onset of depression [8]. There is also an increased risk of bullying and social exclusion, which contribute to low self-esteem and greater psychological vulnerability [9].

The lack of specialized medical services for adolescents with ASD hinders continuity of care, especially during school and professional transitions [10].

### Attention deficit/hyperactivity disorder (ADHD)

Although the core symptoms of ADHD, especially hyperactivity, tend to decrease over time, in up to 60 % of cases they persist into adolescence and adulthood [11]. Impulsivity and inattention may manifest in a less disruptive manner, but they still have a significant impact on academic performance, social relationships, and mental health [12].

The risk of comorbidities such as mood disorders, substance abuse (e-cigarettes) or alcohol consumption, and risky behaviors (e.g., dangerous challenges) increases during this period [13], requiring continuous monitoring and therapeutic reassessments.

### Intellectual disability and other conditions

Adolescents with intellectual disabilities require specific approaches that involve preparation for adulthood, the development of functional skills for some socioeconomic independence, and extended family support [14]. Sexuality and the desire for autonomy also emerge as central topics that need to be discussed sensitively and ethically during consultations with adolescents and their families by pediatricians [15].

### Transition models and integrated care

The transition to adolescence should be planned in a structured manner, focusing on the continuity of medical, educational, and social care. Models based on individualized transition plans (ITPs) have proven effective in preparing adolescents for the adult environment [16].

The collaborative work of pediatricians, psychiatrists, educators, and psychotherapists is essential. International guidelines recommend that the transition process ideally begin between the ages of 12 and 14, focusing on developing self-care and decision-making skills [17]. Periodic diagnostic reassessment of these patients is recommended, considering that clinical manifestations and functional demands may change over time, requiring adjustments to therapeutic strategies [18].

Monitoring adolescents over the years shows that in neurodivergent youth, anxiety symptoms appear before puberty and predict the development of depression in cases with autism spectrum disorder, ADHD, suggesting the need for early intervention [19].

At the same time, it is essential to promote the adolescent's progressive autonomy, encouraging their active participation in therapeutic decisions, which contributes to strengthening their identity and engaging in self-care.

The different interventions range from behavioral strategies to psychosocial, pharmacological, and educational support and should be used in an integrated manner. The choice of the ideal therapeutic strategy depends on the adolescent's specific characteristics, their level of functioning, the presence of comorbidities, and the family and social context in which they are inserted.

The focus of therapeutic approaches should be on promoting the individual's overall development and their autonomy in adulthood. Among the main proposed approaches, the authors highlight behavioral therapy, drug management of comorbidities such as anxiety, psychosocial support through support groups, personalized education with pedagogical adaptations and, more recently, the use of assistive technologies such as applications and virtual reality, in addition to continuous family support [20].

Behavioral therapy is often the starting point for interventions targeting neurodivergent patients, especially those with ASD. Applied Behavior Analysis (ABA) has been widely used in this population, with positive results reported in the scientific literature. This approach involves applying principles of behavior analysis to increase desirable behaviors and reduce problematic or avoidant behaviors through positive reinforcement and other behavioral strategies [21].

Furthermore, developing structured routines is a key element of behavioral intervention. Creating clear routines, with set times for study, social activities, and leisure, can help increase a sense of safety and control over one's environment [22].

Although behavioral and psychosocial interventions are considered first-line treatments for neurodivergent individuals, there are cases in which medication is necessary, especially when associated psychiatric comorbidities such as generalized anxiety, depression, or mood disorders are present. In these cases, the judicious use of medications can be essential to improving the adolescent's quality of life and enabling greater engagement in behavioral and educational therapies. Selective serotonin reuptake inhibitors (SSRIs), such as fluoxetine and sertraline, are frequently used to manage anxiety in adolescents [23].

Another essential aspect of therapeutic monitoring for adolescents with a developmental disorder is psychosocial support. Support groups for these adolescents have proven particularly effective in providing a safe and supervised space for these young people to interact with their peers, share experiences, and develop social skills in a structured manner.

Formal education plays a central role in the life of any adolescent. In the case of neurodivergent individuals, adapting the educational environment and curriculum is essential to ensure effective inclusion and academic achievement. The Individualized Educational Plan (IEP) should go beyond basic literacy or behavioral goals and include long-term objectives, such as preparing for university or vocational entrance exams, inclusion in extracurricular activities, and developing functional learning skills for a first job.

It is important that teachers be trained to understand the particularities of these students, adapting their methodologies, using visual aids, concrete language, tasks with well-defined deadlines, and adapted assessments.

Therapies specifically aimed at individuals with ASD have incorporated technological resources that significantly expand the possibilities for intervention. Assistive technologies in the digital world, such as social simulation apps, modeled videos, and interactive games, help adolescents practice social skills in controlled environments with immediate feedback. Many of these apps are specifically developed to promote skills such as emotion recognition, theory of mind development, and understanding of social rules [24].

Another promising resource is virtual reality (VR), which allows simulating complex social situations—such as a job interview, a classroom presentation, or even a meeting with friends—in a completely safe environment. VR enables the replay of experiences, the personalization of scenarios, and performance analysis, all of which contribute to the generalization of learned skills to the real world [25].

It is essential to emphasize the role of the family in the success of any therapeutic plan. Parents and caregivers are not merely observers of the process; they are active participants and often the main mediators of the strategies proposed in therapy. Therefore, family involvement must be systematic and planned.

## The transition from adolescence to adulthood in individuals with neurodevelopmental disorders

The transition from adolescence to adulthood marks an important point in human growth, characterized by significant transformations in the biological, psychological, and social domains. For those with neurodevelopmental disorders, this phase often brings additional challenges that require special attention from healthcare professionals, educators, and family members.

Although these disorders begin in early childhood, they often continue throughout life. However, their expressions, needs, and consequences change significantly over time. During adolescence, aspects related to independence, social integration, academic performance, and identity formation become more pronounced. The transition to adulthood brings new functional demands that, in many situations, are not fully met by health and education services that are predominantly focused on children.

The symptoms of NDDs can transform as they transition into adulthood. In ASD, for example, although some repetitive behaviors and communication difficulties persist, it is common to observe a refinement in interactional patterns, while new issues such as social anxiety and isolation emerge [26]. In the case of ADHD, longitudinal studies show that hyperactivity tends to decrease with age, while inattention and impulsivity often persist, affecting academic performance and interpersonal relationships [27].

The development of professional and personal autonomy is one of the main goals for people with NDD, and this process is significantly compromised in many cases. Difficulties with emotional self-regulation, time management, and daily tasks directly impact the ability to live independently [28].

Even individuals with good academic and cognitive performance may experience significant deficits in practical daily life skills, such as financial management, personal care, and public transportation.

A study of young adults with ASD showed that only a minority lived independently, held a job, or maintained regular social relationships without additional support [29]. The lack of structured transition programs contributes to this gap, often leaving these young people without support during the most critical phase of their development into adulthood.

Permanence in the educational system and the transition to higher education or the job market are particularly challenging stages. Many adolescents with NDD finish high school with curricular adaptations but encounter barriers in continuing their academic careers or finding jobs compatible with their skills [30].

Inclusive education, while a step forward, still shows shortcomings in preparing students with NDD for the demands of adulthood. The lack of individualized plans that address transition goals, such as career guidance, social skills, and independent living practices, is one of the main criticisms raised by experts [31].

In the employment field, young people with NDD face high rates of unemployment or underemployment. In the case of ASD, for example, studies indicate that <20 % of autistic adults are able to maintain employment in positions compatible with their cognitive and academic capacities [32]. Barriers related to communication, adaptation to unstructured environments, and difficulties in understanding implicit social rules are factors that hinder these individuals' professional integration.

Regarding specialized support, the transition to adulthood often coincides with the reduction or even cessation of specialized services offered during childhood. Many adolescents with NDD no longer receive regular monitoring by multidisciplinary teams when they reach adulthood (at age 18 and, in many cases, at age 21, according to the Child and Adolescent Statute (ECA), which leads to discontinuity in care and worsening of their clinical condition [33].

Furthermore, mental health issues become more prominent at this stage. Symptoms of depression, suicidal ideation, and self-harm are more common in young adults with NDD than in the general population [34]. Factors such as bullying, social isolation, low self-esteem, and difficulties adapting contribute to this situation of concern.

For this reason, it is essential that healthcare systems adopt transition models that ensure continuity of care, with teams prepared to support patients throughout their life cycle. Case management models and coordination between pediatric, adolescent medicine, and adult health services are highlighted as best practices [35].

In this context, families play a central role in the transition to adulthood for young people with NDD. Parents and caregivers often become the primary mediators between the adolescent and the healthcare, education, and employment systems. However, they also face emotional, financial, and physical burdens, especially when there is no effective support network [36].

Cultural and socioeconomic factors also directly influence the autonomy of these young adults. In countries with fewer resources, such as Brazil, or where support services are scarce, the responsibility for care tends to fall entirely on the family, further hindering the development of functional independence [37].

Consequently, public policies should include family support programs, including mental health education, support groups, and legal and financial guidance. Preparation for the future should begin in adolescence, with active parental participation in transition planning.

Several countries have developed specific public policies for the transition of adolescents with chronic conditions and NDD into adulthood. Models such as the UK's "Ready Steady Go" model propose a structured approach, beginning preparation at age 14, with progressive stages leading up to the full transition at age 18 or 21 [38]. This model includes periodic interviews with the young person and their caregivers, the development of individualized plans, and integration with clinical and adult education services.

In Brazil, although there are guidelines for care for people with disabilities, such as the 1999 Disability Inclusion Law (Decree N. 3298 of December 20, 1999, which regulates Law N. 7853/89), and establishes the National Policy for the Integration of Persons with Disabilities, consolidating protection standards and establishing guidelines for the social inclusion and citizenship of this population, there are still few services specifically structured for the transition. The lack of coordination between the health, education, and social assistance sectors compromises the continuity of care. The implementation of specialized care teams for adolescents, focusing on life planning, career guidance, and psychosocial support, is urgently needed.

Another relevant aspect of the transition to adulthood is the development of sexuality and romantic relationships. Many adolescents with NDD have difficulty understanding social norms related to intimacy, consent, dating, and even marriage. At the same time, they often have limited access to sex education, due to family fears or the lack of training of health and education professionals [39].

The lack of adequate guidance increases the risk of violence and abuse, sexual exploitation, and inappropriate behavior. Therefore, it is essential that the topic of sexuality be addressed in an accessible, respectful, and ongoing manner. Sex education programs adapted to the language and cognitive abilities of adolescents have proven effective in promoting safety and autonomy.

The transition to adulthood for individuals with NDD should not be viewed as a one-off event, but as an ongoing process that requires advance planning, multidisciplinary involvement, and family support. Interventions should begin in adolescence, focusing on the development of practical, social, and emotional skills, as well as strategies for educational and professional inclusion.

Advances in research and public policy formulation have contributed to greater awareness of the needs of this population, but there is still a long way to go. The implementation of structured transition programs, professional training, continuity of mental health care, and support for families is are essential tool to ensure that these adolescent adults reach their potential and lead independent, full, and meaningful lives.

## Conclusion

Adolescence for individuals with neurodevelopmental disorders is a phase of multiple challenges and opportunities. Integrated, person-centered, continuous, and collaborative healthcare is essential to promote the well-being and inclusion of these adolescents.

The lack of clear public policies and organized care flows remains a significant obstacle in Brazil. Investments in public policies, professional development and training, and integrated transition models are priorities to improve long-term outcomes.

Promoting integration between pediatric services and adolescent mental health professionals is essential to ensure the full development and quality of life of these young people, a human rights issue guaranteed in Article 227 of the Brazilian Federal Constitution.

## Data availability

The data that support the findings of this study are available from the corresponding author.

## Conflicts of interest

The authors declare no conflicts of interest.
